# How human-derived brain organoids are built differently from brain organoids derived from genetically-close relatives: a multi-scale hypothesis

**DOI:** 10.1039/d5sm01116g

**Published:** 2026-01-19

**Authors:** Tao Zhang, Sarthak Gupta, Madeline A. Lancaster, J. M. Schwarz

**Affiliations:** a Department of Polymer Science and Engineering, School of Chemistry and Chemical Engineering, Shanghai Jiao Tong University Shanghai 200240 China zhangtao.scholar@sjtu.edu.cn; b Department of Physics, Syracuse University Syracuse NY 13244 USA jmschw02@syr.edu; c MRC Laboratory of Molecular Biology, Cambridge Biomedical Campus Francis Crick Avenue Cambridge CB2 0QH UK; d Indian Creek Farm Ithaca NY 14850 USA

## Abstract

How genes influence tissue-scale organization remains a longstanding biological puzzle. While experimental efforts quantify gene expression, chromatin, cellular, and tissue structure, computational models lag behind. To help accelerate multiscale modeling, we demonstrate how a tissue-scale, cellular-based model can be merged with a cell nuclear model incorporating a deformable lamina shell and chromatin to test hypotheses linking chromatin and tissue scales. Specifically, we propose a hypothesis to explain structural differences between human, chimpanzee, and gorilla-derived brain organoids. Recent experiments reveal that a cell fate transition from neuroepithelial to radial glial cells includes a new intermediate state that is delayed in human-derived organoids, leading to significantly narrowed and lengthened apical cells. Additional experiments also demonstrated that ZEB2, a transcription factor, plays a major role in the onset of the novel intermediate state. We hypothesize that this delay stems from chromatin reorganization triggered by mechanical strain as the respective brain organoids develop, with a higher critical threshold in human-derived cells. Here, we computationally test the feasibility of such a hypothesis by exploring how slightly different initial configurations of chromatin, as modeled by different numbers of chromatin crosslinkers, organize in response to mechanical strain with increasingly different initial configurations representing less genetically-close relatives. We find that even small differences in the number of chromatin crosslinkers (>0.01%) yield distinguishable chromatin displacement on average beyond 35% mechanical strain. At higher strains, we observe a new type of nonlinear chromatin scaling law with an exponent of 3.24(5). Finally, we show how differences in chromatin strain maps and more conventional contact maps can reveal structural distinctions between genetically-close species.

## Abstract 2

Our new multiscale model proposes a critical nuclear strain that triggers ZEB2 activation, delayed in human-derived organoids.

## Graphical abstract

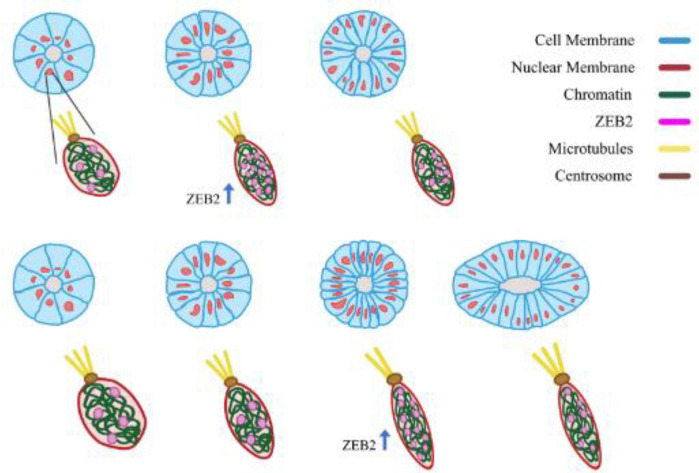

## I. Introduction

Genetic mutations can indeed impact tissue scale organization. For instance, there is plentiful experimental evidence that changes in gene expression can affect the foliated structure of a developing brain.^1–4^ To be even more specific, mutations of the LIS1 gene result in lissencephaly, or a smooth brain.^5,6^ Although the connection between genes and tissue-scale organization is highly complex, with many puzzle pieces still unknown, experimental efforts are actively working to uncover them. Specifically, recent experimental progress on linking the chromatin scale with the tissue scale is now emerging with, for example, the finding that mechanical straining a tissue leads to the loss of heterochromatin to give rise to cell nuclear softening.^7^ Structural measurements at the cell and tissue scale have long been standard in biology. Measurements of the spatial organization of chromatin in cells using chromatin conformation capture techniques are now also well-established.^8–15^ Other methodologies acquiring additional information about chromatin architecture include immunoGAM^16^ and spatially resolving chromatin modifications^17^ will also help put together this puzzle. Given these experimental developments, one wonders how the current state of computational modeling can also help solve this puzzle. This manuscript gives a roadmap on how to begin to build minimal, multiscale computational models to help solve this puzzle.

**Fig. 1:**
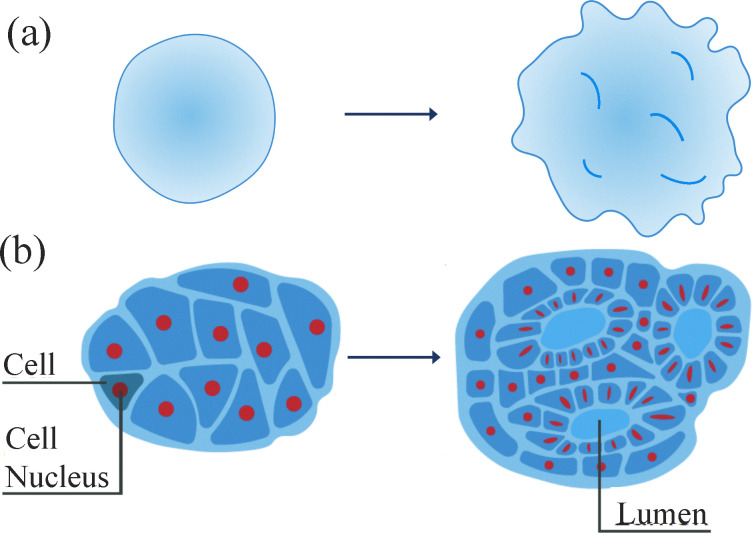
Brain organoid schematics (a) schematic of a brain organoid at an earlier time and at a later time. (b) Schematic of a cross-section of (a) with more cellular detail.

Given some recent, intriguing experimental results on brain organoids,^18^ we will use these results as a guide. As the brain is being built, it is composed of living, multiscale matter capable of emergent forms of mechanical, chemical, and electrical functionality at the genome scale, the cell nucleus scale, the cellular scale, and/or the tissue scale in a nested structure with interplay between the different scales. While such a multiscale materials neuroscience viewpoint may seem obvious-but-unwieldy to many, given the theoretical and experimental techniques that have evolved over the past few decades, we are now on the cusp of being able to develop quantitative predictions based on this viewpoint for the brain structure–function relationship, and, more importantly, to test them using brain organoids^19–21^—an *in vitro* realization of a developing brain. See Fig. 1.

Delving into the experimental findings, even before the onset of neurogenesis, the human forebrain, consisting of precursor cells known as neuroepithelial (NE) cells, is larger than other mammals.^22^ It has, therefore, been long hypothesized that differences in these NE cells may result in expansion of the neocortical primordium.^23,24^ The expansion begins as tangential expansion and then becomes radial as asymmetric NE cell division emerges with one daughter radial glial (RG) cell (and the other daughter a NE cell).^25^ The RG cells do not inherit epithelial features of NE cells and are rather elongated and, presumably, provide patterning for neurons. Since it is difficult to explore this hypothesis in humans and apes, recent experiments study human and ape brain organoids derived from induced pluripotent stem cells (iPSCs).^18^ Intriguingly, human-derived brain organoids exhibit larger surface area than their ape-derived counterparts.^18^ In studying the NE-RG cell transition in such brain organoids, an intermediate cell morphology was discovered and named transitioning NE (tNE) cells. In tNE cells, cell shape changes occur prior to the change in cell identity. This intermediate cell morphology is delayed in human brain organoids in comparison with ape brain organoids. Since the delay in tNE formation postpones the transition from tangential-to-radial expansion, this delay, combined with a shorter cell cycle for human progenitor cells, leads to a larger progenitor pool and, thus, typically larger human-derived brain organoids.

To understand the molecular mechanisms behind this delay in tNE formation in human NE cells, time-resolved sequencing analysis helped to identify differential expression in the zinc-finger transcription factor ZEB2 and, so, a potential driver of tNE cell formation.^18^ ZEB2 as a driver was then tested in mutant ZEB2+/− brain organoids as well as controlling ZEB2 expression such that the human-derived and ape-derived brain organoids achieve a similar size and morphology with, for example, the addition of doxycycline to induce ZEB2 expression at earlier stages. Additional treatments validated this hypothesis.

Given such findings, we now ask how does ZEB2, and potentially other players, regulate the delay in the NE-RG cell transition? To answer this question requires understanding of what lies in a cell nucleus. Transcription factors are proteins that control the rate of transcription of genetic information. Of course, these proteins themselves need to be made and so we must understand what controls their own expression rates. While there are a number of pathways regulating transcription given that the NE-RG transition is dominated by cells elongating and so changing shape, we are going to pursue a means of regulation that is mechanical in nature—mechanical in that some initial cell shape change can potentially induce additional cell shape changes that can potentially lead to chromatin reorganization. Since genetically-close relatives do not have the same chromatin organization, particularly at the topologically-associating domain scale,^26^ it would behoove us to ask how such differences in chromatin organization change as the cell nucleus changes shape. Do the differences become amplified or not, or remain the same? And how do such differences modify gene expression?

Here, we pose a multi-scale hypothesis that may provide a mechanism for delaying the NE-RG cell transition as well as introduce a computational model that can begin to address chromatin reorganization as a function of cell nucleus shape change—functionality that is key to our hypothesis. Specifically, we first state a falsifiable hypothesis linking tissue-scale compression to nuclear deformation and chromatin reorganization relevant to ZEB2 regulation. We then implement a minimal two-component multiscale framework: a 3D vertex model to generate cell-shape dynamics under localized organoid compression, and a deformable nucleus-chromatin mechanics model to quantify chromatin reorganization under mechanical loading. The two components are coupled by using cell-shape-derived compression to prescribe the nuclear loading protocol, enabling a direct test of the feasibility of the hypothesis test within a controlled modeling framework.

## II. A multiscale hypothesis

What do we mean by a mechanical means of regulating transcription? Let us consider human iPSCs. Genetic information is stored in the cell nucleus and when combined with histones, forms chromatin. Chromatin is spatially and temporarily organized within the cell nucleus. While a difference in genetic sequence between, say, a chimpanzee and a human, is rather small—approximately about 1.2%^27^—perhaps even this rather small difference in genetic sequence translates into differences in spatial organization of the genome inside a cell nucleus. Incidentally, Hi–C maps of human *versus* chimpanzee stem cells demonstrate differences.^26^ Such differences in spatial organization of the genome can potentially translate into differences in gene expression dynamics, such as ZEB2. Moreover, the spatial organization of chromatin can be modified by a change in the shape of a nucleus, which is often due to a change in the shape of the cell with cell nuclear shape often mimicking cell shape.^28,29^ As evidence for this, Golloshi *et al.*, study chromosome organization before and after melanoma cells travel through 12 micron and 5 micron constrictions to find compartment switching between euchromatin and heterochromatin, among other differences, when performing the Hi–C analysis.^30^

**Fig. 2:**
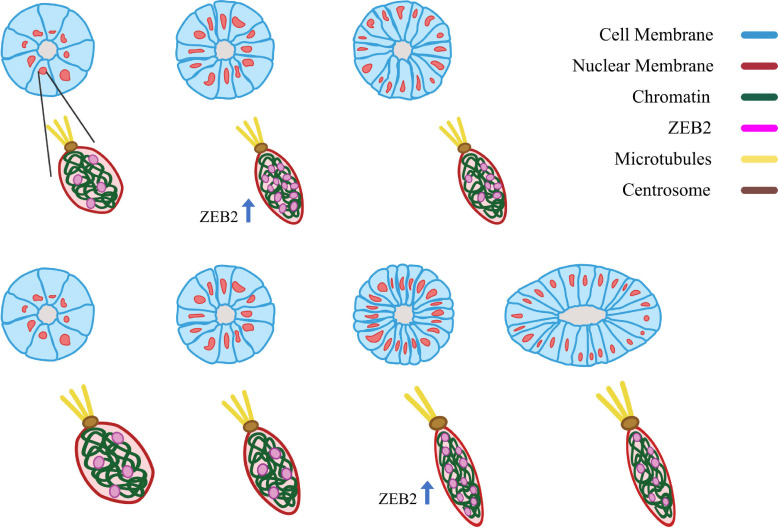
A multiscale hypothesis: the top row is a sketch of the time evolution of the cross-section of one cortex-lumen structure in a gorilla-derived brain organoid. The bottom row depicts the cross-section of the same structure, but in a human-derived brain organoid. According to our multiscale hypothesis, there is a critical strain on a cell nucleus to help initiate upregulation of ZEB2. The critical strain is larger for human-derived pluripotent stem cells as compared to gorilla-, or chimpanzee-derived pluripotent stem cells, resulting in a delay in the human-derived brain organoids. Note that ZEB2 can take on multiple roles, including inhibiting BMP-SMAD signaling by inhibiting BMP4 transcription to disrupt cell–cell junction formation and regulating the production of the microtubule-centrosome binding protein ninein. Note that that all components, particularly ZEB2, are not necessarily to scale. Graphics credit: Savana Swoger.

As the NE cells divide, given the brain organoid is developing in a confined environment, we hypothesize that the additional cells generate compression on, say, a cell of focus. As the compression increases, there is presumably a slight change in cell shape, which may result in a change in nuclear shape, which then may result in a change in the spatial organization of the chromatin. For instance, a slight compression in a particular direction (and hence elongation of the nucleus in the direction perpendicular to the compression), may open up a chromatin region to facilitate/enhance ZEB2 expression. With this enhancement, presumably ZEB2 is able to take on additional functionality. See Fig. 2.

We hypothesize that the amount of compression and/or compression rate required to modify the chromatin organization associated with ZEB2 expression varies from human iPSCs to ape iPSCs. More precisely, a higher amount of compression is needed for human iPSCs as compared to gorilla- or chimp-derived iPSCs.

Once ZEB2 expression increases, there are multiple downstream effects that can impact cell shape. For instance, ZEB2 is a regulator of SMAD signaling that can affect the production of cell–cell junction proteins.^31^ Should the upregulation of ZEB2 lead to fewer cell–cell junction proteins at the apical side, then the cells are able to more readily contract at the apical side. Moreover, the actin-binding protein SHROOM3 helps strengthen the stress fibers oriented in such a way to facilitate constriction.^32^ Manipulation of SMAD signalling resulted in influencing the onset of tNE morphology, while treatment with LPA countered the apical constriction.^18^ In addition to diminishing the strength of cell–cell junctions, enhancing apical constriction, microtubule organization may also be affected. It is known that ZEB2 regulates the production of the microtubule-centrosome binding protein Ninein,^33^ which may help guide the cell fate transition towards a RG cell given that microtubules shape RG morphology.^34^ Moreover, Fouani, *et al.* find that ZEB1 switches from being a transcription factor to a microtubule-associated protein during mitosis.^35^ And while they do not find the same phenomenon for ZEB2, the multi-functionality for this class of proteins is rather intriguing.^36^ In any event, given the downstream changes to cell adhesion and cell cytoskeletal organization to alter the cellular forces at play, the cell shape transition to more elongated cells drives radial-like expansion of the brain organoid.

Our chromatin-reorganization-due-to-cell-compression hypothesis is readily testable using Hi–C at the single iPSC level to determine at what amount of compressive strain does, or does not, alter the chromatin organization pertaining to ZEB2 expression. At the brain organoid level, the mechanical perturbations are self-generated, if you will, by the cells and the influence of the environment in which the brain organoid is embedded. The more cell nuclei become deformed, the more we need their explicit description in cellular-based models. Note that we would like to go beyond the typical biochemical signaling pathways through cell–cell junctions, focal adhesions, or YAP/TAZ by which others have studied nuclear mechanotransduction^37^ to explore directly chromatin organization.

Experimental tests of this multi-scale phenomenon will either validate, or not validate, the hypothesis. Here, we ask the question: How can we build minimal, multi-scale models to computationally generate such hypotheses prior to performing gene expression experiments such that the modeling informs the experiments as opposed to experiments informing the modeling? We argue that several of the multi-scale pieces are coming into focus to allow us to more readily connect genetic-scale processes to tissue-scale processes, though there is still much to do. The pieces that are coming into focus are cellular-based models for the structure of organoids as well as structural models of deformable cell nuclei containing chromatin.^38,39^

We developed a three-dimensional vertex model to predict the early development morphology and rheology of cellular collectives,^38^ representing cells as deformable polyhedrons without gaps, termed confluent. Our model, applied with periodic boundary conditions, reveals a rigidity transition at a critical shape index of 
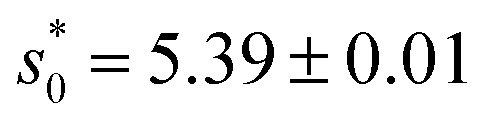
. We observed a distinct boundary-bulk effect in confluent collectives subjected to lateral and radial extensile deformations, with the effect spanning a single-cell layer thickness. Specifically, cells within the bulk align less with lateral deformations compared to boundary cells, indicating that internal cells are largely shielded from deformations over slow timescales and moderate strain. This finding sheds light on cell shape patterning mechanisms in organoids and live organisms. We also developed a computational model at the cell nucleus scale to quantify nuclear mechanics and morphology.^39–41^ The model incorporates activity as well as the deformability of the lamina shell, chromatin-lamina linkages, and chromatin crosslinks. In addition to quantitatively capturing stress–strain curves revealed in experiments, it also provides a new mechanisms for correlated chromatin motion and for nuclear bleb initiation.^39–41^

**Fig. 3:**
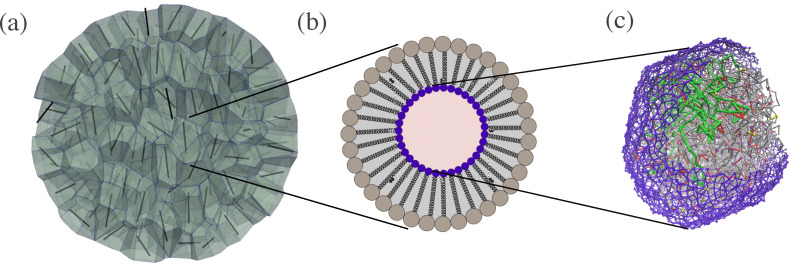
A computational approach to multiscale modeling of tissue structure: (a) a representative organoid based on a three-dimensional vertex model in which cells are represented as deformable polyhedrons and there are no gaps between the cells. The black rods denote the long axis of the polyhedron as determined by a fit to a minimal volume ellipsoid. (b) A schematic of a two-dimensional cross-section of a cell that includes the acto-myosin cortex (outer ring of springs), the lamina shell (inner ring of springs), and the bulk cytoskeleton, including vimentin (the springs connecting the inner and outer ring of springs). (c) A deformable lamina shell cell nuclei (purple) containing chromatin (grey). A portion of the chromatin is colored in green to highlight its configuration.

In the Computational model Section, we detail these two recent computational models: one focused on the tissue scale and the other examining the cell nucleus scale. We will then demonstrate how they can be coupled to continue to probe a key brain organoid structure question that has been recently asked and begun to be answered experimentally: how does the development of the structure of human-derived brain organoids differ from their closest genetic relatives, namely chimpanzee-derived and gorilla-derived brain organoids? We will demonstrate, in principle, how our multiscale hypothesis can be first tested computationally to determine its feasibility. In other words, we provide a roadmap for a direction of next-generation multi-scale, cellular-based computational models that are minimal—minimal in the sense that complexity emerges from simplicity as opposed to complexity emerging from complexity. They can also be falsified by experiments as they yield predictions about organoid shape and rheology, cell shape and rheology and cell nucleus shape and rheology and chromatin structure, ultimately. With falsification, comes progress of some form. See Fig. 3 for an overview of the computational framework that we now detail.

## III. Computational model

### A cellular-based computational model for organoids

A.

Let us begin with a cellular-based, computational model for an organoid that is rooted in earlier work.^42–49^ With such a model, we can track changes in cell shape, which can potentially give rise to changes in nuclear shape such that changes in nuclear shape can potentially lead to changes in chromatin organization. To this end, let us review recent construction of a what is called a three-dimensional vertex model with boundaries.^38^

Cells are biomechanical and biochemical systems that operate out of equilibrium, driven by internal or active forces. The biomechanics of an organoid, which is a cluster of cells, is described by the energy functional:1
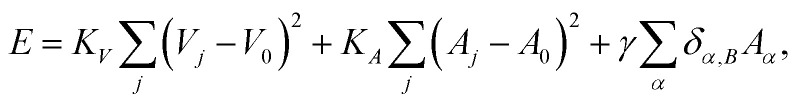
where *A*_*j*_ is the total area of the *j*th cell, *V*_*j*_ is its volume, and *α* represents the faces of the cells. The term *δ*_*α*,B_ is 0 if a cell face is not at the boundary B of the collective and 1 otherwise. The formula includes penalties for deviations from a cell's preferred volume and area, with *K*_*V*_ and *K*_*A*_ being the stiffness coefficients for volume and area, respectively. The volume term reflects the cell's bulk elasticity, with *V*_0_ as the target volume.

The area term, when expanded, includes quadratic, linear, and constant parts. It is typically suggested that the quadratic term relates to the contractility of the actomyosin cortex, while the linear term, with a coefficient of −2*K*_*A*_*A*_0_, results from the balance between cell–cell adhesion and cortical contractility. Negative values, representing smaller *A*_0_, indicate dominance of cortical contractility, and positive values, representing larger *A*_0_, indicate dominance of cell–cell adhesion.

Indeed, cell–cell adhesion and contractility are coupled.^50^ For instance, knocking out E-cadherin in keratinocytes, effectively changes the contractility.^49^ Given this intricate coupling, it may be difficult to tease out the competition. Moreover, the finding in two-dimensional vertex models of a rigidity transition as the target perimeter is increased then leads to the interpretation that unjamming, or fluidity, is given by an increase in cell–cell adhesion, which appears to be counterintuitive.^48^ As for an alternative interpretation, by adding a constant to the energy, which does not influence the forces, again, the energy can be written in the above quadratic form. Since we cannot tune cell–cell adhesion independently of cortical contractility, we posit that the target area is simply a measure of the isotropy of cortical contractility, assuming that curvature changes in the cells remain at scales much smaller than the inverse of a typical edge length. It is the cell–cell adhesion that is bootstrapped to the cortical contractility as cell faces are always shared. To be specific, the larger the target area, the less isotropically contractile the cell is, and *vice versa*. The less isotropically contractile a cell is, the more it can explore other shapes to be able to move past each other in an energy barrier-free manner resulting in fluidity. Additional terms linear in the area for specific cell faces complexify the notion of isotropic contractility. See Table 1 for the parameters implemented in the vertex model.

**Table 1:** Table of the parameters used in the vertex model simulations with the values listed in simulation units

Parameter	Symbol	Value
Active energy	*k*_B_ *T*_eff_	10^−4^
Simulation timestep	*dτ*	0.005
Cell area stiffness	*K*_*A*_	1.0
Cell volume stiffness	*K*_*V*_	10.0
Cell target volume	*V*_0_	1.0
Cell target surface area	*s*_0_	5.0–5.8
Boundary cell surface tension stiffness	*γ*	1.0
Reconnection event threshold edge length	*l*_th_	0.02
Number of cells	*N*	152
Damping	*ξ*	1

Regarding the linear area term in eqn (1), for cells located at the boundary of the organoid, an additional surface tension term is introduced for faces that interact with the “vacuum,” which consists of empty cells. These empty cells do not exert forces on the other cells, but they enable the surface cells of the organoid to relax. Additionally, at this stage, there is a constraint preventing cells from separating from the organoid.

For any length *l* in the simulation, it can be nondimensionalized using the relation *l* = *V*_0_^1/3^. This process highlights a crucial parameter in these models, the dimensionless shape index *s*_0_ = *A*_0_/(*V*_0_^2/3^), which correlates with the target area. For instance, a regular tetrahedron possesses a dimensionless shape index of approximately *s*_0_ ≈ 7.2.

We have addressed the biomechanical aspect of cells with biochemical aspects indirectly encoded into the model parameters. We must also account for their dynamics. Cells can move past each other even when there are no gaps between them. In two dimensions, such movements are known as T_1_ events. Understanding these events are key to understanding the rigidity transition in two dimensions.^48^ In three dimensions, such movements are known as reconnection events. Prior work has developed an algorithm for such reconnection events focusing on edges becoming triangles and *vice versa* that may occur for edges below a threshold length *l*_th_ for a fixed topology.^46^ Specifically, each vertex has four neighboring vertices and shares four neighbor cells. Each edge shares three cells and each face/polygon shares two cells. Our modeling builds on that key work.^46^

In addition to reconnection events, there is an underlying Brownian dynamics for each vertex. Specfically, the equation of motion for the position **r**_*I*_ of a single vertex *I* is

2 **ṙ**_*I*_ = *μ***F**_*I*_ + *μ***F**^B^_*I*_,

with **F**_*I*_ and **F**^B^_*I*_ denoting the conservative force and the random force due to active fluctuations on the *I*th vertex respectively. The force **F**_*I*_ is determined from both the area and volume energetic constraints and, hence, includes cell–cell interactions. Moreover, each vertex performs a random walk with an effective diffusion coefficient of *μk*_B_*T*_eff_, where *T*_eff_ is an effective temperature due to activity. Unless otherwise specified, the mobility *μ* = 1. To map from simulation units to biophysical units, one simulation time unit maps to 50 seconds, one simulation length unit maps to 10 microns, and one simulation energy unit maps to one *k*_B_*T*_eff_ Finally, the Euler–Maruyama integration method is used to update the position of each vertex.^51^

### B. A computational model of deformable cell nuclei

The cell nucleus houses the genome, or the material containing instructions for building the proteins that a cell needs to function. For humans and other genetically-close relatives, this material is ∼1 meter of DNA. Using proteins to form chromatin, the DNA is packaged across multiple spatial scales to fit inside an ∼10 µm nucleus.^52^ In addition, chromatin is highly dynamic; for instance, correlated motion of micron-scale genomic regions over timescales of tens of seconds has been observed in mammalian cell nuclei.^53–57^ This correlated motion diminishes both in the absence of ATP, the fuel for many molecular motors, and by inhibition of the transcription motor RNA polymerase II, suggesting that motor activity plays a key role.^53,54^ These dynamics occur within the confinement of the cell nucleus, which is enclosed by a double membrane and 10–30-nm thick filamentous layer of lamin intermediate filaments to form a lamina shell.^58–60^ The lamina shell is deformable and, as such, one can quantify its shape fluctuations. Specifically, depletion of ATP diminishes the magnitude of the shape fluctuations, as does the inhibition of RNA polymerase II transcription activity.^61^

Chromatin and the lamina shell interact directly *via* lamina-associated domains (LADs)^62,63^ and indirectly through various proteins.^64–66^ Therefore, the spatiotemporal properties of chromatin can potentially influence shape of the lamina shell and *vice versa* as the two components are coupled. Indeed, studies have found that depleting linkages between chromatin and the nuclear lamina, or membrane, results in more deformable nuclei,^67,68^ enhanced curvature fluctuations,^69^ and/or abnormal nuclear shapes.^70^ Another recent study suggests that inhibiting motor activity diminishes nuclear bleb formation.^71^ Moreover, depletion of lamin A in several human cell lines leads to increased diffusion of chromatin, suggesting that chromatin dynamics is also affected by linkages to the lamina.^72^ Together, these experiments demonstrate the critical role of chromatin and its interplay with the lamina shell in determining nuclear shape.

To quantify chromatin dynamics and nuclear shape, we constructed a chromatin-lamina system with the chromatin modeled as an active Rouse chain and the lamina as an elastic, polymeric shell with linkages between the chain and the shell. We also included chromatin crosslinks, which may be a consequence of motors forming droplets^73^ and/or complexes,^74^ as well as chromatin binding by proteins, such as heterochromatin protein I (HP1).^75^ Recent rheological measurements of the nucleus support the notion of chromatin crosslinks,^76,77^ as does indirect evidence from chromosome conformation capture (Hi–C).^15^ Unlike previous chain-enclosed-by-a-deformable-shell models,^69,76,77^ our model has motor activity. We implemented the simplest type of motor, namely extensile and contractile monopoles that act non-reciprocally on the chromatin.

To be even more specific, interphase chromatin is modeled as a Rouse chain consisting of *N* monomers with radius *r*_c_ connected by Hookean springs with spring constant *K* (Table 2). We include excluded volume interactions with a repulsive, soft-core potential between any two monomers, *ij*, and a distance between their centers denoted as |*r⃑*_*ij*_|, as given by3
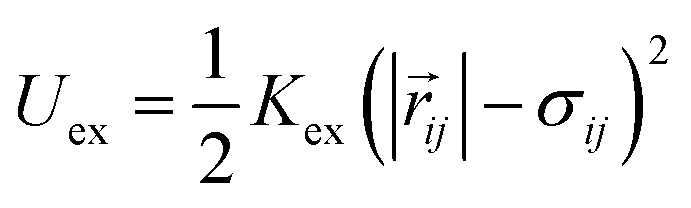
for |*r⃑*_*ij*_| < *σ*_*ij*_, where *σ*_*ij*_ = *r*_*c*_*i*__ + *r*_*c*_*i*__, and zero otherwise. We include *N*_C_ crosslinks between chromatin monomers by introducing a spring between different parts of the chain with the same spring constant as along the chain. In addition to Gaussian fluctuations, we also allow for explicit motor activity along the chain. To do so, we assign some number, *N*_m_, of chain monomers to be active. An active monomer has motor strength *M* and exerts force **F**_a_ on monomers within a fixed range. Such a force may be attractive or “contractile,” drawing in chain monomers, or alternatively, repulsive or “extensile,” pushing them away. Since motors *in vivo* are dynamic, turning off after some characteristic time, we include a turnover timescale for the motor monomers *τ*_m_, after which a motor moves to another position on the chromatin.

**Table 2:** Nucleus model parameters in simulation units used throughout, unless otherwise specified

Parameter	Symbol	Value
Chromatin monomers	*N*	5 × 10^3^
Spring constant	*K*	140
Soft core spring	*K*_ex_	140
Number of crosslinkers	*N*_C_	2440–2500
Lamina monomers	*M*	10^4^
Number of linkages	*N*_L_	400
Diffusion constant	*D*_*T*_eff__	1
Mobility	*μ*	1

The lamina is modeled as a layer of *M* monomers connected by springs with the same radii and spring constants as the chain monomers and an average coordination number *z* ≈ 4.5, as supported by previous modeling^69,76,77^ and imaging experiments.^58–60^ We modeled the chromatin-lamina linkages as *N*_L_ permanent springs with stiffness *K* between shell monomers and chain monomers (Fig. 3(c)). There is an additional soft-core repulsion between monomers making up the lamina shell to include excluded volume.

The system, as is the case for the three-dimensional vertex model, evolves *via* Brownian dynamics, obeying the overdamped equation of motion:

4 *ξ***ṙ**_*i*_ = (**F**_br_ + **F**_sp_ + **F**_ex_ + **F**_a_),

where **F**_br_ denotes the Brownian/Gaussian force, **F**_sp_ denotes the harmonic forces due to chain springs, chromatin crosslink springs, and chromatin-lamina linkage springs, and **F**_ex_ denotes the force due to excluded volume. The magnitude of the Gaussian force is 
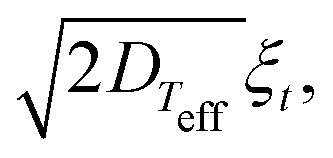
 where *ξ*_*t*_ is a Gaussian variable with zero mean and unit variance.

Finally, we used the same simulation parameters as in ref. 41 with a varied number of crosslinks as specified below, though motor activity was turned off. To implement the compression, we applied a boundary condition at the top and bottom of the nucleus, where we moved the boundary at a specified strain rate. If a lamina particle is beyond the boundary, it experiences a spring force towards the boundary such that the lamina surface is compressed due to the moving boundary. Converting from simulation units to biophysical units, we used the following mapping: one simulation length unit is 1 µm, one simulation time unit is 50 s, and our simulation energy unit is *k*_B_*T*_eff_ ≈ 10^−21^ J. In these units, the spring constant is 1.4 × 10^−4^ nN µm^−1^ and our simulation runs for 5 × 10^4^ s (about 14 h) with a strain rate of 8 × 10^−6^ s^−1^ to compress the nucleus to 60% of its original size, with 20% on each side. Ten realizations are obtained for each parameter varied.

### C. Coupling the organoid model to the cell nucleus model

Now that we have two of the major players in the process—cells and cell nuclei, let us now envision how we can embed one into the other. Earlier work has embedded nuclei in single cells in two-dimensions to understand cell motility in confinement.^78^ In that work, the cell nuclear cortex is connected to the cell cortex *via* springs modeling the remainder of the cellular cytoskeleton, including vimentin, beyond the cell cortex. See Fig. 3(b). The model is, therefore, more detailed than prior minimal models, while still remaining foundational in that it reveals a new cell polarity mechanism regulated by vimentin. Given that a direct embedding of cell nuclei of every cell using springs, as was done in the two-dimensional confined cell motility study discussed above, is somewhat detailed even in two dimensions, we will do something simpler. We will instead make the simplifying assumption that nuclear shape tracks cell shape such that any changes in cell shape result in changes in nuclear shape. For instance, should a cell become elongated, its cell nucleus will also become elongated with the same strain. This assumption is supported by a number of prior experiments.^28,29,79–81^

To begin to computationally test the notion that chromatin organization can change in response to tissue structure in development, we start with a structure that is reminiscent of a dominant brain organoid structure after several days in development (approximately Day 5) in terms of a collection of cells surrounding a central lumen structure. A lumen is a fluid-filled structure. We used the same simulation parameters as in ref. 38, but with fewer cells—152 cells, due to the presence of the lumen. Over the course of several days, the cells in a brain organoid have divided. Given the confinement of the environment, the dividing cells become compressed in various directions. Here, to study the change in shapes of cells as the organoid undergoes compression, we compressed cells in a ring-shaped area in the center of the organoid inward by driving the outer vertices radially inward at a strain rate of 20% over a time interval of 1000 simulation units. While our compressive strain is external, for organoids confined within Matrix gel and undergoing cell division, the compressive strains generated are internal.

## IV. Results

**Fig. 4:**
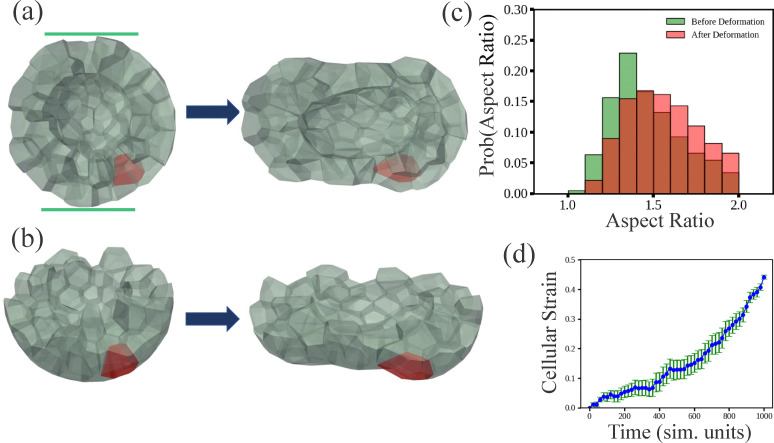
Localized compression of a model brain organoid containing a lumen: (a) top view of before and after compression a fluid-like brain organoid with *s*_0_ = 5.6 and cut in half to better expose the interior. The compression occurs in the region denoted by the green lines. (b) Side view of (a). (c) Probability distribution of the aspect ratio of each cell before and after the applied compression/deformation. (d) Plot of the cell undergoing the largest compressive strain (thickness reduction along the final-time short-axis direction) as the organoid undergoes compression, averaging over 20 realizations. The corresponding cell is labelled in red in (a).

We apply external compression to our model brain organoid, as discussed in Section 3. We do this for organoids with a target shape index of *s*_0_ = 5.6. See Fig. 4. In these simulations, we compressed cells in a ring-shaped area in the center of the organoid inward by driving the outer vertices radially inward. In the deformed configuration, we observe that cells in the vicinity of the compressed ring exhibit shape changes that are well-described, to leading order, as a predominantly uni-axial compression (thickness reduction along a principal axis). We therefore quantify the cellular compression using a thickness-based compressive strain. Specifically, for each cell we fit its vertex positions at the final time *t*_f_ to a minimal-volume ellipsoid to determine the principal axes and define **n̂**_s_ as the corresponding short-axis direction. Using this fixed direction, we compute the cell thickness at any time *t* from vertex projections, *H*(*t*) = max_*i*_[**r**_*i*_(*t*)·**n̂**_s_] − min_*i*_[**r**_*i*_(*t*)·**n̂**_s_], where **r**_*i*_(*t*) are the cell vertex positions. We then define the compressive engineering strain as *ε*_c_(*t*) = [*H*(0) − *H*(*t*)]/*H*(0), which is positive under compression. In Fig. 4(a) and (b), the cell colored red denotes the cell with the largest final compressive strain *ε*_c_(*t*_f_). In Fig. 4(c) we plot the probability distribution of the aspect ratio of cells before and after the deformation. As a result of the deformation, the probability distribution becomes more biased toward cells with higher aspect ratios. Since applied strain is what one can control in the simulations, as opposed to shape, in Fig. 4(d) we also plot *ε*_c_(*t*) as a function of simulation time for the cell in red in Fig. 4(a) and (b), averaging over 20 realizations. We will use this time-dependent cellular compressive strain (and its strain rate) to prescribe a corresponding uni-axial compression protocol in the nuclear simulations below.

Now that we have the relationship between changes in cell strain, which is related to cell shape, in response to tissue-scale compression, we use a simplifying assumption to proceed at the next smallest length scale. Again, we assume that cell nuclear shape tracks cell shape.^28,29,79–81^ We then take mechanical deformations that track cell deformations and apply them to our deformable cell nucleus to ask how does the chromatin organization change? While our model for chromatin is a coarse-grained one, we can still ask the following questions for several types of perturbations: for a given number of chromatin crosslinks and linkages, by how much does the chromatin chain locally displace in the presence of uni-axial compression? And should we perturb the number of crosslinks or linkages by how much does the local displacements change? We will use small differences in the number of crosslinkers as a minimal model for slightly different chromatin structures to represent genetically-close species in which the pairwise geonomic interactions remain conserved, however, higher order chromatin structure, such as topologically-associated domains, appear not be conserved.^26^ Specifically, the reference human state is 0 chromatin crosslinkers removed (*N*_C_ = 2500) and that the genetically less close relatives consist of increasingly more chromatin crosslinkers removed. Our generic chromatin model can teach us something about how the chromatin reorganizes in response to cell nuclear shape changes given cellular shape changes.

**Fig. 5:**
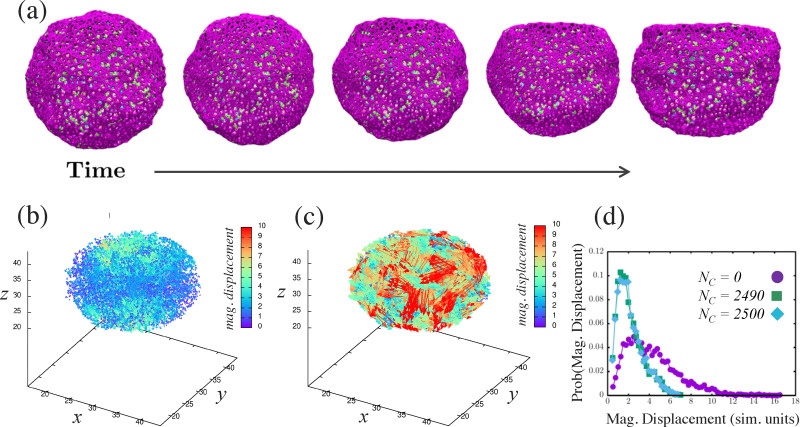
Compressing a model cell nucleus consisting of a lamina shell containing chromatin: (a) snapshots of the model cell nucleus being compressed for number of chromatin crosslinks *N*_C_ = 2500 and number of linkages *N*_L_ = 400. The strain rate is the same as in Fig. 4(d). From left to right, the snapshots are at *t* = 1, 250, 500, 750, 1000, respectively. (b) Plot of the chromatin displacement field for (a). (c) Plot of the chromatin displacement field for *N*_C_ = *N*_L_ = 0 for comparison. (d) Probability distribution of the magnitude for the chromatin displacement field comparing (b) with (c) and also for *N*_C_ = 2490, *i.e.*, a small change from the configuration in (a). For (b) and (c) the data is for the final simulation time (*t* = 1000).

Given the time-dependent compressive strain (thickness reduction rate) of the maximally compressed cell, we apply the corresponding uni-axial loading protocol to a model cell nucleus and measure the displacement of chromatin monomers, as demonstrated in Fig. 5. In Fig. 5(a), we show snapshots from uni-axial compression of the lamina shell by two parallel plates moving at constant speed towards each other and exerting force on the lamina shell, but not on the chromatin. We can do so for the same initial chromatin configuration, though with different number of chromatin crosslinks and linkages. Naturally, for a larger number of chromatin crosslinks, one expects smaller displacements. Indeed, we observe smaller displacements for a larger number of chromatin crosslinks with *N*_C_ = 2500 and *N*_L_ = 400, compared with the other extreme with *N*_C_ = *N*_L_ = 0, as shown in Fig. 5(b) and (c). In Fig. 5(d) we plot the probability density function of the magnitude of the displacements for the two cases in Fig. 5(b) and (c), and confirm the fact that the more crosslinked chromatins displaces less. Note that we have only considered one type of perturbation from the reference state here. For now, we have turned off activity, which could be yet another generator of changes in gene expression for two genetically very similar genomes.

In what follows, if chromatin were purely a liquid on both short and long spatial scales and time scales, such a comparison between a reference state and a perturbed state in terms of a genetically-close relative under applied compression, as is done here, may not provide much insight. However, experiments demonstrate that the presence of chromatin crosslinks establish the need for a more intricate rheology of chromatin such that it can act elastically over some time and length scales.^82–84^ For instance, mechanical measurements of stretched cell nuclei can be readily explained with chromatin crosslinks.^82^ And recent isotropic swelling of cell nuclei reveal very reproducible chromatin configurations before and after the swelling.^84^ Other experiments indicate that a Burger's model fits micropipette aspiration data.^85^ Moreover, linkages in terms of LADs between chromatin and the lamina shell also potentially provide some elasticity over shorter time scales.^39^ Presumably, linkages dominate closer to the periphery than in the bulk. Given the complex rheology of chromatin, we perform the following analysis.

**Fig. 6:**
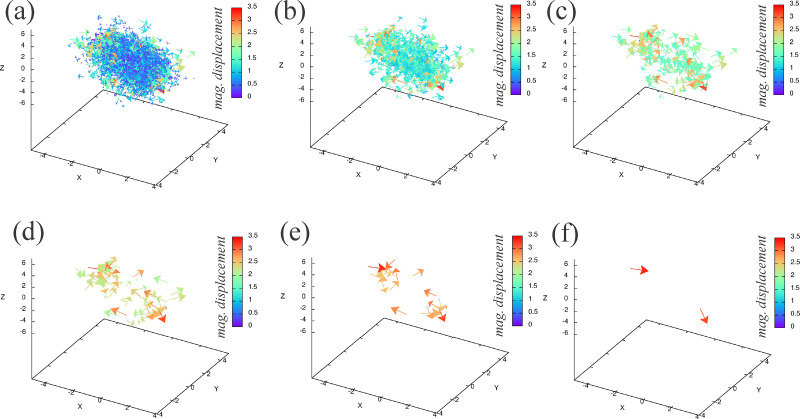
Comparing differences in chromatin displacement fields between the unperturbed and perturbed chromatin configuration with 0.4% change in the number of chromatin crosslinkers: (a)–(f) the threshold magnitude of the difference in chromatin displacements between *N*_C_ = 2500 and *N*_C_ = 2490 increases from top left to bottom right. By increasing the threshold magnitude, from 0.0 to 1.0 to 1.5 to 2.0 to 2.5 to 3.0 in simulation length units in (a)–(f) respectively, one can more readily identify regions of differences. These regions of differences do not necessarily correspondence to the differences in locations of the chromatin crosslinkers.

We now determine whether or not a small change in the number of chromatin crosslinks (for the same initial chain configuration) will lead to pockets of differences in displacements in the chromatin. Such pockets could be candidates for changes in genetic expression, even within this minimal model. In Fig. 5(d), we plot the probability density function for 10 fewer chromatin crosslinks, for the same initial configuration, and find small differences in the distribution from the unperturbed case. We then study the differences in displacements between the largest number of crosslinks and the perturbed case with slightly fewer crosslinks, keeping in mind that with Brownian dynamics, there will always be some difference in the structure. We are looking for differences above that noise floor. The spatial map of the differences in displacements for one realization (Fig. 6) does exhibit pockets of larger differences in displacements with such regions being candidates for differences in gene expression with the assumption that changes in chromatin configuration can potentially influence genetic regulatory networks at the base pair level. Those pockets become more pronounced the larger the threshold magnitude of displacements.

**Fig. 7:**
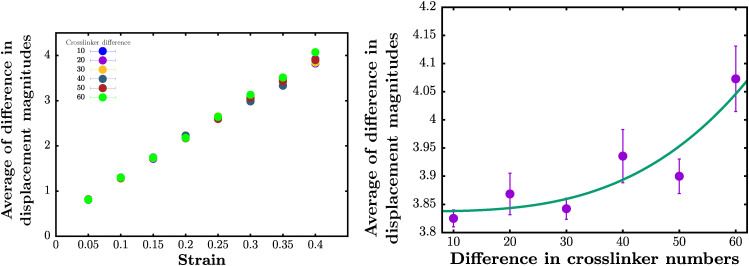
Difference in magnitude of the total average chromatin displacement for different numbers of crosslinkers for different strains: left: plot of the difference in the average of the magnitude of displacements between *N*_C_ = 2500 and the number of removed chromatin crosslinkers for different values of the strain. Note that not until approximately 35% strain can one distinguish between the different numbers of chromatin crosslinkers. Right: Plot of the difference in the magnitude of the total average chromatin displacement between *N*_C_ = 2500 and *N*_C_ = 2490 (difference in crosslinker number equals 10) and between *N*_C_ = 2500 and *N*_C_ = 2480, (difference in crosslinker number equals 20), *etc.*, for 40% strain. The green curve is the result of a power law fit with an additional constant. The power law fit yields faster than linear behavior with a power of *α* = 3.24(5). The units of displacement are in terms of chromatin monomer radius.

While Fig. 6 shows the differences for one realization, we also quantify the difference in the magnitude of the total average displacement between the fully crosslinked case (*N*_C_ = 2500) and smaller numbers of crosslinks (given the same initial configuration of both the lamina shell and the chromatin) for 24 realizations (see Fig. 7). First, we study the difference in the magnitude of the total average displacement between different species as a function of the amount of applied strain (Fig. 7 (left)). For strains up to ∼30%, the curves of chromatin reorganization for different numbers of crosslinkers collapse and are numerically indistinguishable when focusing on the larger differences, *i.e.*, when the total average displacement exceeds the threshold of 3.5 monomer radius (Fig. 7 (left)). Distinguishable differences emerge only beyond ∼35% strain, where a statistically significant separation begins to appear.

Given the differences across species by 40% strain, we focus on that value of the applied strain and now plot the difference in the magnitude of the total average displacement *versus* increasingly different species (see Fig. 7 (right)). We find that as the two configurations to be compared are increasingly different numbers of crosslinks, that the difference in the magnitude of the total average displacement increases. This result is not necessarily surprising. However, does this difference diverge exponentially with slightly different initial configuration? If yes, then perhaps the system is chaotic. One would not necessarily anticipate this trend as such behavior would presumably hamper the nucleus to carry out its functionality in a robust manner. After fitting several different phenomenological forms, we find that for *y* denoting the difference in the magnitude of the total average displacement and *x* denoting the difference in number of crosslinkers,

5 *y* − *b* ∝ *x*^*α*^

with *α* = 3.24(5) and *b* = 3.84(3) reasonably approximates the data. In other words, the difference increases faster than linear. Given the complex chromatin rheology, a nonlinear scaling is not necessarily surprising. As increasingly different numbers of crosslinkers are placeholders for increasingly different species, then obtaining such quantities as *α*, even phenomenologically, will help further elucidate comparisons between them at the chromatin scale. This nonlinear scaling form is a novel type of scaling form that should be studied across species.

**Fig. 8:**
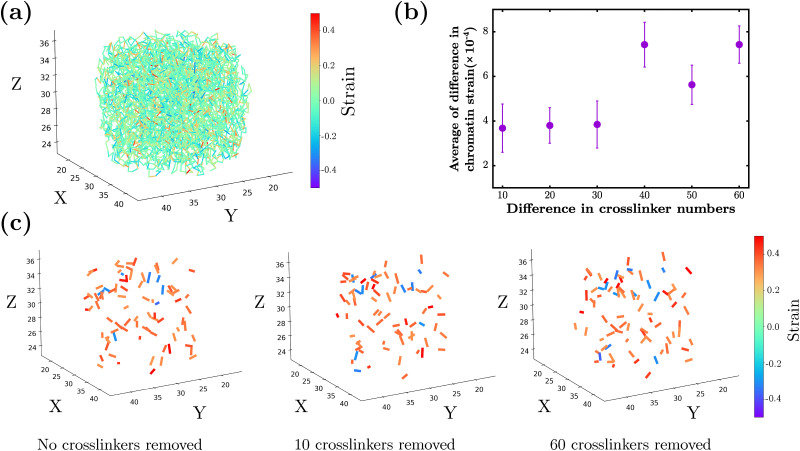
Differences in strains for different numbers of crosslinkers: (a) strain map for *N*_C_ = 2500 for one realization at the end of the applied compression. (b) Plot of the average difference in chromatin strain between *N*_C_ = 2500 and fewer chromatin crosslinks. (c) Strain maps at the end of the applied compression for *N*_C_ = 2500 (0 removed), *N*_C_ = 2490 (10 removed), and *N*_C_ = 2440 (60 removed); chromatin springs are shown only when |*ε*| > 0.30. The color bar denotes the chromatin spring strain *ε* (dimensionless) over the range *ε* ∈ [−0.5, 0.5].

We go beyond the differences in displacement of chromatin to also quantify the differences in strain. In Fig. 8(a), the strain map for the spring between each chromatin monomer is plotted for *N*_C_ = 2500 for a typical instance. While most of the springs contain very small amounts of strain, there are springs whose magnitude of strain is greater than 30% with positive strain, or springs under tension, being favored. Just as in Fig. 7, we also plot the average of the difference in strains between *N*_C_ = 2500 and subsequently smaller numbers of crosslinkers for 24 realizations (see Fig. 8(b)). This average increases with the increasing difference, though the trend is more difficult to quantify than the average of the difference in magnitude of chromatin monomer displacements. However, as the chromatin configurations become more different given the increasing difference in number of crosslinkers, the average of the difference in strain tends to increases, as expected. After 40 crosslinkers are removed, a statistically significant difference from the noise floor emerges. When examining strain maps for a particular sample depicting a magnitude of strain greater than 30%, there is a bias towards positive strain for *N*_C_ = 2500, 2490, and 2440 (Fig. 8(c)). Moreover, there appears to be a spatial localization of large negative strain springs that is somewhat conserved for the three cases. It is also interesting to note with more crosslinkers removed, there is an increase in the number of chromatin springs under large tension.

**Fig. 9:**
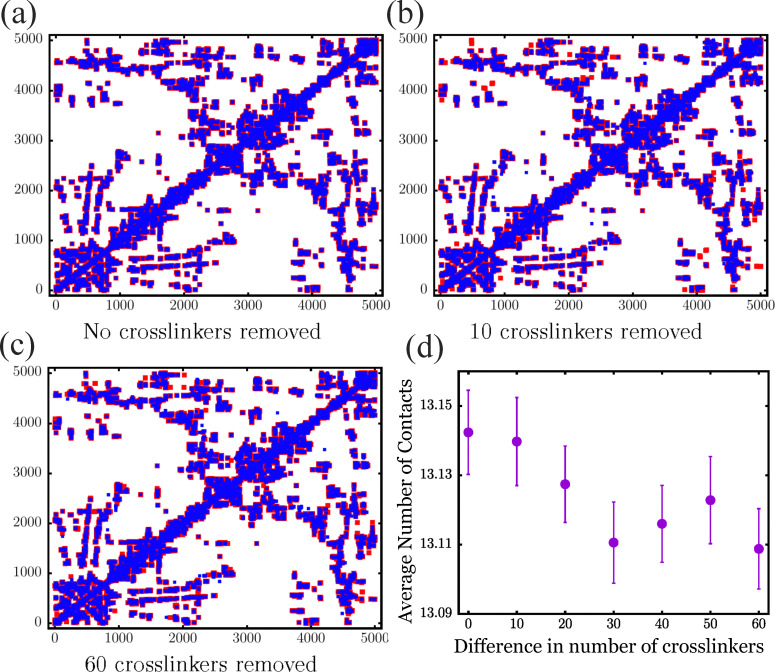
Differences in chromatin contacts for different numbers of crosslinkers: (a)–(c) contact maps for different numbers of chromatin crosslinkers, *N*_C_ = 2500, comparing *N*_C_ = 2500 with *N*_C_ = 2490, and comparing *N*_C_ = 2440 with *N*_C_ = 2500, respectively. The red squares in (b) and (c) denote differences between the two types of configurations. (d) Plot of the average number of contacts per chromatin monomer as the number of crosslinks decreases for chromatin configurations at 40 percent strain and for the final simulation snapshot.

To make contact with more conventional chromatin configuration measures, Fig. 9(a)–(c) show the contact map, or adjacency matrix, when two particles are in contact or not with 2 chromatin particle diameters. If they are in contact, they are colored; otherwise, they are not. For *N*_C_ = 2500, the particles in contact are denoted in red (Fig. 9(a)); for the removed crosslinker cases, the particles in contact are denoted in blue and are slightly larger squares (Fig. 9(b) and (c)). We can therefore overlay removed crosslinker cases with no crosslinker removed case, so we can see where there are differences. For *N*_C_ = 2500 case, the two maps perfectly overlap, as they should, but for 10 and 60 crosslinkers removed, there are some purely red squares and some purely blue squares demonstrating the effect of eliminating crosslinkers. These regions should correlate with the differences in displacement maps shown in Fig. 5 and 6 as differences in displacements can lead to changes in neighbors. Fig. 9(d) demonstrates that the average number of contacts per particle decreases with fewer crosslinkers as expected.

## V. Discussion

We posit a testable, multi-scale hypothesis for a difference in brain organoid structures derived from human-derived pluripotent stem cells and chimpanzee-derived pluripotent stem cells during the first ten days of development. The hypothesis involves, ultimately, mechanical perturbations of cell nuclei with human-derived pluripotent stem cells demonstrating a different critical strain for particular regions of chromatin organization as compared to chimpanzee-derived pluripotent stem cells. The particular regions of chromatin organization are relevant to changes in gene expression of the ZEB2 transcription factor that can ultimately impact cell shape by way of decreasing apical cell adhesion and increasing apical cell constriction, as demonstrated previously.^18^

While experimental confirmation awaits, we ask what insights can computational modeling provide in terms of building such testable, multi-scale hypotheses. So here we focus on a minimal, falsifiable multiscale hypothesis-testing framework, rather than a fully coupled multiscale model with feedback. We argue that using cell-based, computational modeling in terms of, for example, a three-dimensional vertex model as presented in ref. 38 is a reasonable starting point for an organoid, particularly during the early stages of development. Such models can ultimately provide accurate descriptions of cell shape. To link the cell scale with the cell nuclear scale, we make the simplifying assumption that nuclear shape tracks cell shape.^28,79–81^ And now that there exists a coarse-grained mechanical model for a chromatin-containing cell nucleus that allows for deformability and that recapitulates nuclear mechanics, correlated chromatin motion, and changes in nuclear bleb initiation due to the addition of α-amanitin,^39,41,76^ we can begin to study, at some level, chromatin reorganization in response to mechanical deformations. Other chromatin-based models do not yet allow for nuclear deformability or account for the lamina shell.^55,86–89^

Since our focus is to look at structural differences between genetically-close species whose chromatin organization is very similar at smaller pair-wise scales but not necessarily at larger scales,^26^ we determine how chromatin organization differs in response to mechanical deformations between a reference state and a perturbed state with fewer chromatin crosslinks for the same initial chromatin configuration as a case study. We denote the reference state as human state and the perturbed state as the genetically-close relative. We find that as strain is increased on the cell nucleus, it is only at larger strains that statistically significant differences between the reference and the perturbed states emerge. Moreover, at larger strains of, for example, 40%, we find a faster than linear scaling relation between increasingly less genetically-close relatives and the average difference in displacements between the reference (or human) chromatin state and the genetically-close chromatin state. Such a finding motivates the need to quantify differences in spatiotemporal chromatin reorganization across species just as comparative anatomy has done over centuries for the brain and many other organs.^90,91^ Future work with enhanced statistics will make such scaling relations more precise and, therefore, enhance the links between tissue structure and chromatin organization.

To obtain more accurate results, one can compute the radius of gyration tensor for the vertices of each cell relative to a reference configuration to quantify the full three-dimensional deformation, and then impose the corresponding deformation on the lamina shell. We leave this for future work. And yet, one must go beyond the cell nuclear shape tracks cell shape assumption. For example, there is experimental evidence for feedback between cell shape and nuclear shape, with the nucleus releasing calcium should it be compressed above a critical strain.^92^ As calcuim promotes the vimentin filament polymerization, perhaps there is added mechanical protection for the nucleus, in addition to actin cytoskeletal mechanism.^92^ We have not yet explored such feedback. Thus, a natural next step is to explicitly embed a deformable nucleus inside each vertex-model cell and mechanically couple the nuclear envelope to the cell cortex through a fiber/spring network representing the intervening cytoskeleton (*e.g.*, vimentin- and actin-mediated connections). This construction would transmit cell-generated, time-dependent deformations to the nucleus in real time, enabling the nuclear shape to evolve directly from the tissue-scale dynamics rather than being prescribed by an imposed loading protocol. It would also provide a practical route to incorporate general 3D deformations (including shear and triaxial modes) and potential mechanical feedback between nuclear and cellular mechanics.

Moreover, since we are ultimately after a predictive model for changes in chromatin configuration as a function of mechanical and chemical perturbations, the cell nucleus model will require more detail such as heterochromatin *versus* euchromatin and liquid–liquid phase separation of chromatin crosslinkers^93^ as well as a more accurate motor representation to work towards predictive Hi–C in the presence of mechanical perturbations, particularly as condensin II appears to determine genome architecture across species.^94^ Efforts are already underway *via* HiCRes and HiCReg that are rooted in libraries, or other means, but do not appear to focus on nuclear shape.^95,96^ Moreover, cell division plays an important role and so understanding how chromatin reorganization during cell division is important as well.

A further limitation is that we do not explicitly address timescale interdependence between the imposed strain rate and chromatin relaxation. In the nuclear simulations we apply a prescribed compression history at a fixed strain rate, without systematically varying loading rate or quantifying rate-dependent reorganization. Because chromatin is viscoelastic, the observed displacement/strain patterns may depend on whether deformation is slow or fast relative to internal relaxation, motivating future strain-rate scans and protocol variations to establish the regime in which strain-amplified differences become observable.

As experimental scientists are able to obtain more detailed information at multiple scales in living systems, it behooves the non-experimental scientists to be able to stitch the scales together not just retroactively but proactively, to be able to better understand the design principles of life. In other words, connecting the dots between genes and tissues theoretically is becoming increasingly within our reach. While here we focused on a multi-scale hypothesis for the structure of brain organoids, one can obviously think more broadly to organoids and tissues in general. Other modelers have begun to realize the importance of multiscale mechanical modeling with a two-dimensional vertex model containing rigid nuclei^97^ and a powerful new three-dimensional mechanical model for cells that also includes nuclei.^98^ Both of these newer models do not yet include chromatin. Moreover, during *in vivo* brain development, mechanical forces are also at play *via* the emerging vascular system that impose additional strains.^99^ These additional strains could influence chromatin organization. Of course, certain types of questions regarding the structure of *in vitro* brains do not require as detailed an explicit framework containing chromatin and so we continue to work on answering them,^100–102^ though not losing sight of the more detailed, multi-scale, computational modeling road that we are just beginning to travel. We hope that many will join us along the way.

## Conflicts of interest

There are no conflicts to declare.

## Data Availability

Data for this article, including codes and simulation raw data are available at Zenodo at https://doi.org/10.5281/zenodo.17557600.
